# Plant resistome profiling in evolutionary old bog vegetation provides new clues to understand emergence of multi-resistance

**DOI:** 10.1038/s41396-020-00822-9

**Published:** 2020-11-11

**Authors:** Melanie Maria Obermeier, Wisnu Adi Wicaksono, Julian Taffner, Alessandro Bergna, Anja Poehlein, Tomislav Cernava, Stefanie Lindstaedt, Mario Lovric, Christina Andrea Müller Bogotá, Gabriele Berg

**Affiliations:** 1 Institute of Environmental Biotechnology, Graz University of Technology, Petersgasse 12/I, 8010 Graz, Austria; 2 ACIB GmbH, Krenngasse 37/II, 8010 Graz, Austria; 3 Genomic and Applied Microbiology and Göttingen Genomics Laboratory, Institute of Microbiology and Genetics, Georg-August University of Göttingen, Grisebachstrasse 8, 37077 Göttingen, Germany; 4 Know-Center GmbH, Research Center for Data-Driven Business & Big Data Analytics, Infeldgasse 13/VI, 8010 Graz, Austria

**Keywords:** Environmental microbiology, Metagenomics, Microbial ecology

## Abstract

The expanding antibiotic resistance crisis calls for a more in depth understanding of the importance of antimicrobial resistance genes (ARGs) in pristine environments. We, therefore, studied the microbiome associated with *Sphagnum* moss forming the main vegetation in undomesticated, evolutionary old bog ecosystems. In our complementary analysis of culture collections, metagenomic data and a fosmid library from different geographic sites in Europe, we identified a low abundant but highly diverse pool of resistance determinants, which targets an unexpectedly broad range of 29 antibiotics including natural and synthetic compounds. This derives both, from the extraordinarily high abundance of efflux pumps (up to 96%), and the unexpectedly versatile set of ARGs underlying all major resistance mechanisms. Multi-resistance was frequently observed among bacterial isolates, e.g. in *Serratia*, *Rouxiella, Pandoraea, Paraburkholderia* and *Pseudomonas*. In a search for novel ARGs, we identified the new class A β-lactamase Mm3. The native *Sphagnum* resistome comprising a highly diversified and partially novel set of ARGs contributes to the bog ecosystem´s plasticity. Our results reinforce the ecological link between natural and clinically relevant resistomes and thereby shed light onto this link from the aspect of pristine plants. Moreover, they underline that diverse resistomes are an intrinsic characteristic of plant-associated microbial communities, they naturally harbour many resistances including genes with potential clinical relevance.

## Introduction

The risk posed to modern medicine by increased morbidity and mortality associated with antibacterial resistance continues to escalate globally and has reached a stage where a post-antibiotic era is not unthinkable anymore [1, 2]. Many of the clinically relevant antimicrobial resistance genes (ARGs) originate from the environment, wherein they may act in intra-community signalling and metabolic processes; in the presence of selective pressure they can adapt antibiotic resistance as primary function [3]. In order to retrace the origin and habitat transitions of resistant microorganisms, a detailed understanding of native resistomes is crucial [3]. So far, such elucidations focused on soil, water and air [4]. Limited work has been performed on plants and thereby mostly revolved around fresh produce to assess the risk potential of crops in serving as gateway of ARGs to humans [5–7]. The resistome of native plants from pristine vegetation was neglected so far. It can, however, provide the missing ecological link to understand the evolution and functioning of native resistomes as well as their role as pools of unexplored resistance mechanisms [8]. Since the resistome reflects the continuous co-evolution of small bioactive molecules and microbial genomes within an environment [9], we expect native plants, which provide an extraordinarily diversified secondary metabolism, to possess a diversified intrinsic resistome as well.

*Sphagnum spp*. covering peatlands, was selected as a model plant to study ARGs in a representative pristine as well as evolutionary old ecosystem [10, 11]. *Sphagnum-*dominated peatlands constitute balancing and productive ecosystems, in which the prevailing harsh conditions fostered symbiotic connections throughout a long plant-microbe co-evolution [12, 13]. As a result, the *Sphagnum* microbiome is highly abundant and diverse with a specialised structure and similar functioning across geographic locations [14, 15]. Furthermore, in *Sphagnum-*dominated bogs mosses together with other bog inhabiting plants and lichens share a core microbiome forming a transkingdom metacommunity [16]. The microbiota fulfils important functions like nutrient supply and protection against biotic and abiotic stress; its metagenome is characterised to a remarkably high extent by signatures indicating horizontal gene transfer and communication systems thought to facilitate the balance between plasticity and stability within the bog ecosystem [13]. Moreover, the highly stable microbiome is not affected by soil microbiota, given that the rootless *Sphagnum* moss grows on peat; accumulated, partly degraded plant material mostly stemming from the plant itself and forming the largest terrestrial carbon sink on Earth [11]. *Sphagnum* mosses harbour specific and rich metabolite profiles [13] and their associated microbiota is characterised by a high proportion of antimicrobial activity [17]. Altogether, *Sphagnum* spp. represent an ideal model to elucidate the antibiotic resistance background of plants which we expect to: (i) comprise predominantly resistances against natural antibiotics due to the missing selective pressure by synthetic ones, (ii) encompass versatile but evenly distributed ARGs due to the diverse and stable microbial community, (iii) contain yet-unknown resistance genes. For our study, we investigated different bog ecosystems across Europe (Austria, Germany, Norway, Sweden), and pursued a unique approach combining analysis of culture collections, in silico data mining of deep-sequenced metagenomic data, and functional metagenomics; the importance of combinatorial approaches for functional validation of in silico predictions was emphasised but rarely considered before [5, 18].

## Methods and materials

### Experimental overview and study sites

**Fig. 1:**
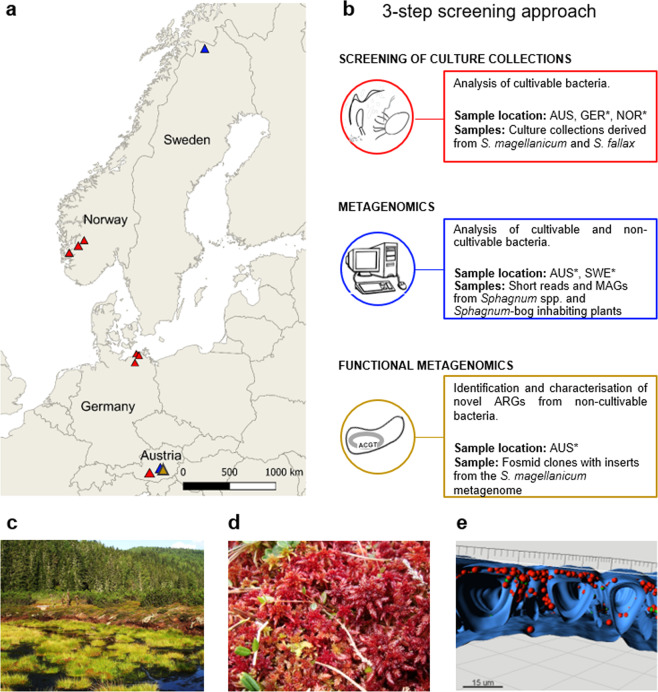
The *Sphagnum*-dominated peat bog. **a** Different bogs in Norway (NOR), Sweden (SWE), Germany (GER) and Austria (AUS), where isolates (red triangles) and metagenomic DNA for the in silico analysis (blue triangles) and the functional metagenomics screening (yellow triangle) derive from. **b** Overview of the three-step screening approach with goal, sampling location and plant species (*Sphagnum fallax*, *Sphagnum magellanicum*). Previously obtained samples are marked with an asterisks. **c** The Austrian Alpine peat bog Pürgschachen Moor. **d** *S. magellanicum* gametophytes. **e** Cross-section of a *Sphagnum* gametophyte displaying the highly abundant microbial colonisation (red spheres) of the moss´s hyaline cells (blue).

For the three-step analysis, a newly obtained culture collection from the Austrian Pürgschachen bog was used together with previously established culture collections from Germany and Norway [13, 17], Austrian and Swedish metagenomes [12, 19, 20] and a metagenomic library derived from Austria [21] (Fig. 1a and b). Employed samples originate from different *Sphagnum* species and other *Sphagnum*-bog inhabiting plants in order to facilitate a more holistic understanding of the peatbog resistome. More detailed information on this as well as sampling dates, locations and references for further details on the sampling procedure for the isolates and metagenomic datasets can be found in Supplementary Data 1 and 2, respectively.

### Sampling, isolation and annotation of *Sphagnum*-associated bacteria from Austria

Gametophytes of the moss *Sphagnum magellanicum* were collected from the Austrian Alpine bog Pürgschachen Moor (N47°34′50.57″ E14°20′29.29″) in September 2017 (Fig. 1c). They were cleaned with sterile tweezers from other plant material, fractionated into sterile plastic bags to batches of 20 g and fragmented with sterile scissors to isolate phyllosphere and endophytic bacteria. Subsequently, 50 ml of chilled 0.85% NaCl solution was added and the moss material was homogenized in a Stomacher laboratory blender (BagMixer, Interscience, Saint-Nom-la-Bretéche, France) twice for 120 s. After straining the suspension through double-layered gauze and a sterile analysis sieve (mesh size 63 µm, Retsch, Haan, Germany), the undiluted suspension as well as serial dilutions thereof were plated on R2A agar (Roth, Germany) containing nystatin (25 µg ml^−1^, Duchefa Biochemie, Haarlem, Netherlands) and incubated at 20 °C for four days. Isolates were subcultured until purity and liquid cultures grown from single, isolated colonies in Nutrient Broth II (Sifin diagnostics, Berlin, Germany) supplied with glycerol to 20% (v/v) for long term storage at −70 °C.

To annotate multi-resistant isolates, cells were mechanically disrupted by bead-beating (6 m s^−1^, 20 s) and the lysates incubated at 95 °C for 10 min followed by centrifugation at 5000 rpm for 5 min. The 16 S rRNA gene was amplified using 2 µl of the supernatant and the universal bacterial primer pair 27 f (5′AGAGTTTGATCMTGGCTCAG) and 1492r (5′TACGGY TACCTTGTTACGACTT), 0.5 µM each, in a 50 µl PCR reaction with 1x Taq-&GO Ready-to-use PCR Mix (MP Biomedicals, Eschwege, Germany) (98 °C − 4 min; 25 cycles of 98 °C - 30 s, 48 °C - 30 s, 72 °C - 90 s; 72 °C - 5 min). The 1400-bp DNA fragments were purified (Wizard®SV Gel and PCR Clean-Up System, Promega, Walldorf, Germany) and sequenced using the same 27 f and 1492r primers in a bidirectional sequencing approach. Sequences were annotated according to their best NCBI database hit.

Fluorescent in situ hybridization and confocal laser scanning microscopy were performed on *Sphagnum* gametophytes as described previously using the reported probes (Cy3-labelled ALF968 for *Alphaproteobacteria*, Cy5-labelled EUB338, EUB338II and EUB338III for *Eubacteria*) [22]. The three-dimensional images and video clip were reconstructed using Imaris 7.0 (Bitplane, Zürich, Sitzerland).

### Antibiotic resistance screening of culture collections and UMAP analysis

Bacterial isolates were screened against ten different antibiotics as listed in Supplementary Table 1. Concentrations were based on those used in other studies [6, 23], which followed the guidelines by the Clinical Laboratory Standards Institute. This included 266 bacterial isolates from Austria, and in total 90 isolates from Germany and 81 from Norway (Supplementary Data 1). All isolates were transferred to R2A agar plates of up to 50 isolates per plate and incubated at 20 °C for 4 days. Colonies were replica printed onto Müller-Hinton agar plates supplemented with the different antibiotics and incubated at 20 °C. The plates were monitored every 24 h for 3 days. This was done in duplicate and only isolates which had grown to visible colonies in both screenings were considered resistant.

Analysis of isolates based on their resistance profile was performed by using Uniform Manifold Approximation and Projection for Dimension Reduction (UMAP) [24, 25] and K-means clustering. To understand discriminative factors in the clusters we trained a random forest classifier [26] with the antibiotics being the predictive features and calculated feature importance on the cluster labels [27]. The relationships between antibiotic resistance clusters and locations were further visualized using a modified version of Caleydo StratomeX [28].

### CARD-based resistome profiling of metagenomic short reads and MAGs

The *Sphagnum* bog metagenomes generated with Illumina sequencing were aligned against protein sequences from the Comprehensive Antibiotic Resistance Database (CARD) [29], retrieved in April 2017. The DIAMOND protein aligner v0.9.24 [30] was implemented and only the forward reads of the paired-end sequenced metagenomes were used to this end. High stringency with a similarity threshold of 90% over the full read length was applied with otherwise default settings. Only hits with ≥30 amino acids identity were retained and manually curated for annotation redundancy or in case of antibiotic target genes for known resistance conferring mutations (Supplementary Table 2). The reads were normalised by calculating the ARAI (antibiotic resistance abundance index: number of reads assigned to one ARG per number of total reads and respective ARG length) [31]. Abundance of ARGs or resistance mechanism within the metagenome was calculated in ppm (≙read per million reads), while their proportion among all assigned ARGs or resistance mechanisms is given as percentage.

SingleM and GraftM were used to calculate abundance of operational taxonomy units (OTUs) and to obtain taxonomical information of the OTUs from each shotgun metagenome dataset [19, 32]. Two marker genes i.e., *rplC* and *S5* were used as indicators to calculate alpha and beta diversity. Phyloseq, and vegan R packages implemented in RStudio were used to analyse bacterial communities and resistome composition [33–36]. Alpha diversity (richness) was calculated for the bacterial communities as well as for the resistomes using the Shannon diversity index (H’) based on normalized datasets. Differences in bacterial community and resistome richness were analysed using the Kruskal Wallis test. Linear regression analysis was used to describe the correlation between bacterial richness and resistome richness. Bacterial community and resistome beta diversity (composition) were calculated based on the normalized Bray-Curtis dissimilarity matrix. A permutational analysis of variance (PERMANOVA) using the adonis function was performed to assess differences in bacterial communities and resistome composition. The correlation between bacterial communities and resistome composition was estimated by the Mantel test [37].

Metagenome-assembled genomes (MAGs) were reconstructed from the Austrian *Sphagnum*-bog metagenomes. In order to reconstruct a high number of high-quality MAGs, the Austrian *S. magellanicum* metagenome from Pirker Waldhochmoor was used independently due to its high sequencing depth, while the other Austrian metagenomes were pooled to represent the overall bacterial community of *Sphagnum*-dominated bogs [16]. Illumina-generated metagenomes were de novo assembled using MEGAHIT with meta-sensitive parameter [38]. The metagenomic contigs were then binned using Maxbin2 v2.2.7, MetaBAT2 v2.12.1 and CONCOCT v1.1.0 [39–41] and dereplicated into MAGs using DASTool v1.1.1 [42]. MAG completeness and the percentage of contamination was calculated using CheckM v1.0.13 [43]. The reconstructed MAGs were combined and analysed together with MAGs that were previously reconstructed from Swedish bog metagenomes [19]. Only MAGs with a completeness of 90% and contamination <10%, that are defined as high quality MAGs according to the current definition of the minimum information metagenome-assembled genome standards [44], were selected for further analyses.

Prodigal was used to predict open reading frames of each MAG. The resulting protein-coding sequences were annotated with DIAMOND [45] against the CARD database [29]. Reads were defined as ARG-like reads at the cut-off of an *E* value of 10^−10^ and similarity of 50% indicating homologous ARGs as previously described [46]. After curation of the hits as described above, the respective protein sequences were retrieved and annotated against eggNOG v5.0 [47]. Protein sequences which were not annotated by eggNOG were assigned as follows: When only a protein identifier was given by eggNOG, annotation occurred through the STRING database v11 [48]. In case of no annotation at all, the respective protein sequences were annotated against NCBI non-redundant protein database. Results were classified as the “same ARG” if CARD and eggNOG assigned the same ARG and as “different ARG” when similar ARGs (e.g., mdtB - mdtC) or another ARG (e.g., mdtA – MuxA) was assigned.

For the in-depth analysis of the *S. magellanicum* metagenome from the Alpine peat bog Pirker Waldhochmoor, RAWGraphs [49] was used to visualise the detected non-efflux pump determinants. The distribution and abundance network of assigned β-lactamases was constructed with Cytoscape v3.3.0 [50].

### Identification of novel antibiotic resistance genes from metagenomic clones

For the functional metagenomics screening, a previously generated 3.6 Gbps fosmid library was employed [21]. Briefly, ~40 kb metagenomic DNA fragments from a *S. magellanicum* dominated, Alpine peat bog were cloned into *E. coli* EPI300 pCC2FOS (Epicentre, Wisconsin, USA). The generated 90 000 metagenomic clones were pooled by resuspending them in LB medium supplied with 20% glycerol for long term storage at −70 °C.

Metagenomic clones were screened against nine different antibiotics as listed in Supplementary Table 1. Concentrations were chosen according to those used in other studies employing the CopyControl system with *E. coli* EPI300 [51, 52]. Alternatively, the minimal inhibitory concentration (MIC) was determined according to the guidelines of the European Committee for Antimicrobial Susceptibility Testing using the broth microdilution method [53]. The assays were conducted in triplicate using the empty vector library host *E. coli* EPI300 pCC2FOS.

The functional metagenomics screening was carried out on LB agar plates containing one of the nine antibiotics and additionally chloramphenicol (12.5 µg ml^−1^) for fosmid maintenance and arabinose (0.01% w/v) to induce high-copy number. Cells of the pooled library stock were revived in LB broth containing chloramphenicol (12.5 µg ml^−1^) at 37 °C for 3 h with shaking at 130 rpm. The library was screened with at least 3× coverage by plating 50,000 to 100,000 CFU per plate. Colonies that had formed after 16 h of incubation at 37 °C were re-cultivated under the same conditions to confirm the phenotype. Resistant clones were evaluated by restriction digest and unique clones were retransformed to confirm the presence of the resistance phenotype on the fosmid insert.

For the identified ampicillin resistant clones, their MIC for ampicillin was determined as described above. For *E. coli* EPI300 pCC2FOS-Mm3 MICs were additionally determined for cefotaxime, cephalothin, cephalexin, carbenicillin (Sigma-Aldrich, Taufkirchen, Germany) using *E. coli* EPI300 pCC2FOS as control strain.

For gene identification de novo sequencing of pCC2FOS-Mm3 was performed. Extracted DNA was used to generate Illumina shotgun paired-end sequencing libraries, which were sequenced with a MiSeq instrument and the MiSeq reagent kit version 3, as recommended by the manufacturer (Illumina, San Diego, California, USA). Quality filtering using Trimmomatic version 0.36 [54] resulted in paired-end reads with an average read length of 301 bp. The assembly was performed with the SPAdes genome assembler software version 3.10.0 [55], resulting in a 50.2 kb contig with a 9.2-fold coverage. The assembly was validated, and the read coverage determined with QualiMap v2.1 [56]. Automatic gene prediction was performed using the software tool Prokka v1.12 [57].

The phylogenetic analysis for inferring the evolutionary relationship of Mm3 with other β-lactamases was conducted using the software MEGA X v10.0.2 [58]. The amino acid sequences were aligned using MUSCLE [59] and the tree was constructed by the neighbour-joining method [60] with a bootstrap test of 2000 replicates, using the p-distance method [61] for computing evolutionary distances.

### Subcloning, expression and characterisation of *blaMm3*

The *blaMm3* gene was cloned into the pET28a(+) expression vector (Novagen, Durham, North Carolina, USA) with N-terminal His Tag and inducible T7 promoter using the NdeI and EcoRI restriction sites. With primers comprising the respective restrictions sites (underlined) (F: 5′–3′ TGCAGACATATGAACCCCAACCACTCTG, R: 5′–3′ TACTAGAATTCCTAGACGCTCGATGTCGCC, Sigma-Aldrich, Germany), the full ORF was amplified from pCC2FOS-Mm3 by a standard PCR reaction using the Phusion DNA polymerase (New England BioLabs, Frankfurth am Main, Germany) at 72 °C annealing temperature. The vector ligated gene was transformed into high efficiency *E. coli* DH5α (New England BioLabs) for selection of the recombinant pET28a-*blaMm3* plasmid, which was then introduced into *E. coli* BL21(DE3) (Thermo Scientific, Waltham, Massachusetts, USA) for overexpression.

For heterologous expression, LB broth (400 ml) with kanamycin (50 µg ml^−1^) was inoculated with 2% (v/v) of an overnight culture of *E. coli* BL21(DE3) pET28a-*blaMm3* which was then grown at 37 °C under shaking at 130 rpm to an OD_600_ of 0.8. The culture was supplemented with isopropyl β-d-1-thiogalactopyranoside to 0.4 mM end concentration and further incubated for 4 h. Harvested cells were resuspended in 50 ml binding buffer (20 mM sodium phosphate buffer, 500 mM NaCl, 20 mM imidazole, pH 7.4) containing 0.8 g l^−1^ lysozyme and disrupted by sonication with a digital sonifier (pulses of 2 s and 4 s pause, 5 min, 70% amplitude; Branson, Emerson, Missouri, USA). The His-tagged protein was isolated from the centrifuged lysate (12000 × *g*, 10 min) using a 1-ml HisTrap column (GE health Care, Chicago, IL, USA) and an elution gradient (1 ml min^−1^, 20 min) up to 500 mM imidazole. Two fractions containing active β-lactamase, as judged by application on nitrocefin disks (Sigma Aldrich) and SDS-PAGE were mixed together. The buffer was exchanged with 20 mM sodium phosphate buffer (200 mM NaCl, pH 7.4) through multiple dilution and centrifugation steps using Amicon Ultra-15 centrifugal filters (10 kDa cuttoff, Merck Millipore, Darmstadt, Germany). Protein purity was estimated with SDS-PAGE and the concentration determined with the Pierce BCA assay kit (Thermo Scientific) using bovine serum albumin as reference. The purified enzyme was shock-frozen in liquid nitrogen and stored at −70 °C.

To determine kinetic values (V_max_ and K_m_) the activity of the purified β-lactamase Mm3 was measured spectrophotometrically (U-2001, Hitachi, Tokyo, Japan). Initial hydrolysis rates for ampicillin and carbenicillin were recorded at 235 nm and 30 °C in 450 µl reaction buffer (20 mM sodium phosphate buffer, 0.2 M NaCl, pH 7.4) upon addition of 50 µl substrate at different concentrations (1 mM to 100 mM in H_2_O). The kinetic data was fitted for ampicillin with the Hill equation and for carbenicillin with the Michaelis-Menten equation, respectively, using the software Origin 9.0.0 G (Origin Lab Corporation, Wellesley Hills, Massachusetts, USA).

### Data accessibility

The Austrian metagenomes are stored at the MG-RAST server: *S. magellanicum*, Pirker Waldhochmoor (4533611.3), *Polytrichum strictum* (4550991.3), *Pleurozium schreberi* (4550992.3), *Sphagnum angustifolium* (4550993.3), *Vaccinium myrtillus* (4550994.3), *Sphagnum fuscum* (4550995.3), *S. magellanicum* (4550996.3), *Eriophorum vaginatum* (4551107.3), *Calluna vulgaris* (4551108.3), *Vaccinium oxycoccos* (4551109.3), *Pinus mugo* (4551110.3), *Andromeda polifolia* (4551111.3) (all Rotmoos), and *Mylia anomala*, Pürgschachen Moor (4551112.3). The MAGs that were reconstructed from the Austrian *Sphagnum*-bog metagenomes can be downloaded from the European Nucleotide Archive under the BioProject PRJEB39100. The Swedish *Sphagnum-*associated metagenomes (short reads and reconstructed MAGs) can be downloaded from NCBI under the BioProject PRJNA386568 [19].

The nucleotide sequence of the β-lactamase *blaMm3* is deposited in Genbank under the accession no. MK831000 and the 16 S rRNA sequences from the multi-resistant bacteria under the accession numbers MK801238 - MK801243 and MN928714 - MN928775.

## Results

### *Sphagnum* isolates display predominantly resistance against (semi)synthetic antibiotics

To visualise the association and colonisation of bacteria with *Sphagnum* mosses, confocal laser scanning microscopy was performed showing dense bacterial populations in *Sphagnum* sp. samples (Fig. 1e, Supplementary Video 1).

**Fig. 2:**
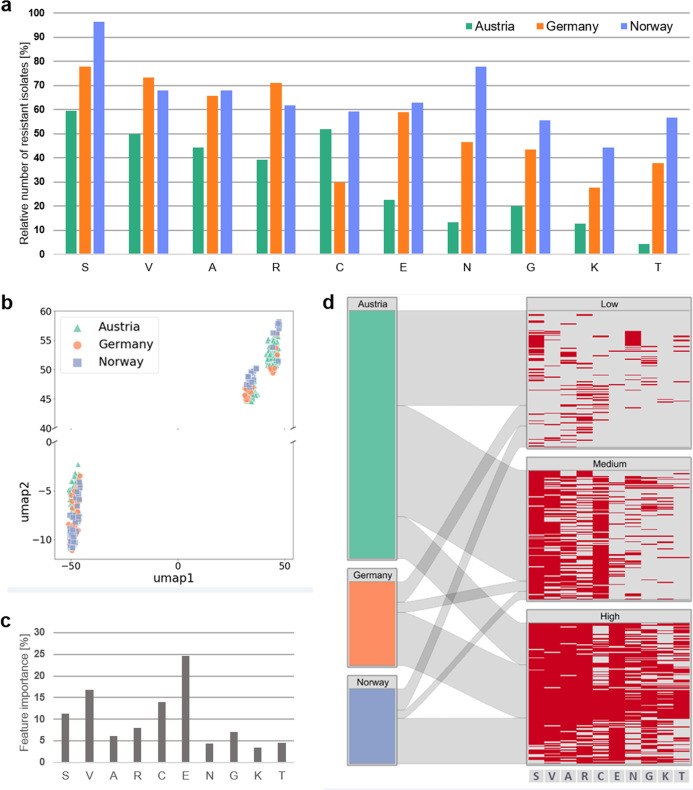
Antibiotic resistance profile of culture collections from *Sphagnum* spp. **a** Relative number of resistant bacterial isolates from *Sphagnum* mosses in Austria, Norway and Germany, which grew in the presence of different antibiotics. **b** Isolate clusters were obtained by the UMAP dimensionality reduction technique with K-means clustering. **c** Feature importance for each antibiotic that determines the clustering in the UMAP analysis. **d** Antibiotic resistance clusters (right) and corresponding locations (left). Blocks on the left show the number and distribution of isolates for each location. Blocks on the right show the clusters labelled according to the number of resistances: low (median = 1), medium (median = 4) and high resistance (median = 7). The heat maps for each cluster give an overview of the antibiotic resistance pattern (red: resistant, grey: not resistant) for sulfadiazine (S), vancomycin (V), ampicillin (A), rifampicin (R), ciprofloxacin (C), erythromycin (E), nalidixic acid (N), gentamycin (G), kanamycin (K) and tetracycline (T).

To evaluate the resistance potential of the cultivable share of *Sphagnum* microbiota, culture collections obtained from *S. magellanicum* and *Sphagnum fallax* gametophytes in Austrian, German and Norwegian bogs (Fig. 1a–d) were screened for resistance against 10 different antibiotics (Supplementary Data 1), comprising those ranked as critically important for medical applications [62]. Overall, resistance against all ten antibiotics was encountered, whereby 88% of the 437 tested bacteria grew in the presence of at least one antibiotic. Except for the natural antibiotic vancomycin, a predominance of resistances against the semisynthetic and synthetic antibiotics sulfadiazine, ampicillin, rifampicin, and ciprofloxacin was observed (Fig. 2a). This contradicts our preliminary expectation of dominating resistances against natural antibiotics.

To assess whether the resistance profiles depend on the geographic location or the microenvironment (*Sphagnum* species), they were compared by means of UMAP analysis. This showed, that the isolates cluster into three separate groups depending on the number of resistances, but independent of the geographic origin (Fig. 2b, c) and neither was the clustering driven by the microenvironment (*Sphagnum* species). The feature importance determining the three clusters, was highest for erythromycin (25%), followed by vancomycin (17%), ciprofloxacin (14%) and sulfadiazine (11%) (given that each antibiotic represents an individual feature) (Supplementary Data 1). This shows that erythromycin was the most important determinant for discriminating between low and high resistance profiles in this dataset. Multi-resistance against eight, nine and ten antibiotics was encountered for 71 isolates across the different bogs (Supplementary Data 1). Most of these isolates were identified as *Serratia* spp., *Rouxiella* sp*., Paraburkholderia* spp., and *Pseudomonas* spp. Other identified genera were *Pandoraea* or *Obesumbacterium* (Supplementary Data 1).

### Bog metagenomes share similar antibiotic resistance traits and comprise a highly diverse resistome

**Fig. 3:**
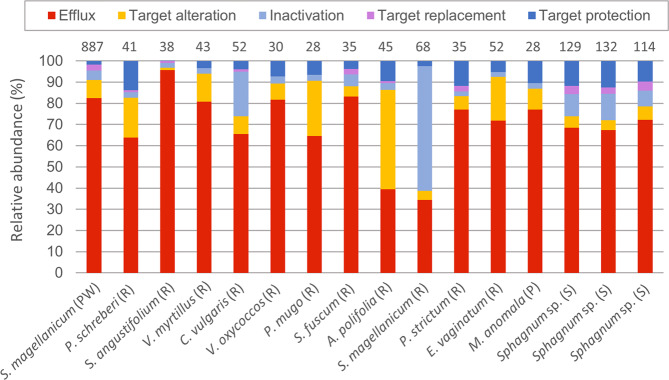
Relative abundance of resistance mechanisms for *Sphagnum-*dominated bogs. The relative abundance of each resistance mechanism is displayed for different Austrian and Swedish metagenomes: antibiotic efflux, inactivation, target alteration, target protection and target replacement. The metagenomes from different bogs in Austria (Rotmoos [R], Pirker Waldhochmoor [PW], Pürgschachen Moor [P]) include *Sphagnum magellanicum*, *Polytrichum strictum*, *Pleurozium schreberi*, *Sphagnum angustifolium*, *Vaccinium myrtillus*, *Sphagnum fuscum*, *Eriophorum vaginatum*, *Calluna vulgaris*, *Vaccinium oxycoccos*, *Pinus mugo*, *Andromeda polifolia*, *Mylia anomala* and from the Swedish Stordalen bog (S) *Sphagnum* sp. The absolute number of detected ARGs is displayed above of each bar.

As only a small proportion of *Sphagnum* microbiota is cultivable, the resistome was analysed through in silico analysis of metagenomes to capture the microbiome at a wider scale including non-cultivable bacteria. Therefore, Illumina-sequenced metagenomic short reads from different *Sphagnum* dominated bogs in Austria and Sweden (Fig. 1a and b, Supplementary Data 2) were aligned against sequences from the CARD using high stringency (90% threshold). This revealed a low abundant, but highly diverse pool of resistance determinants. After curation and double normalisation of matches with ≥30 amino acids identity (Supplementary Table 2), 0.16% of all metagenomic reads were assigned to 943 different ARGs showing even distribution in all metagenomes with abundances ranging from 4.8 × 10^−7^ to 1.5 × 10^−2^ ppm (Supplementary Data 2). A detailed list of all assigned ARGs and their abundance is provided in Supplementary Data 2. The relative number of assigned reads and detected ARGs per Gbps varied across the different metagenomes, showing no direct correlation with sequencing depth. Almost all genetic determinants that were suggested by Berendonk et al. [8] as potential indicators to survey the antibiotic resistance status in environmental samples were detected in these metagenomes, although at low abundance. These include *sul1*, *sul2*, *bla*_CTX-M_, *bla*_TEM_, *bla*_VIM_, *bla*_KPC_, *qnrS*, *vanA*, *mecA*, *ermB*, *ermF*, *tetM* and *aph* (Supplementary Data 2). ARGs related to all major resistance mechanisms were detected: antibiotic efflux, inactivation, target alteration, and target protection were found in all independent samples and target replacement was present in one third of the analysed metagenomes (Fig. 3, Supplementary Fig. 1, Supplementary Data 2). In terms of both, absolute number of ARGs and relative abundance (Fig. 3, Supplementary Fig. 1, Supplementary Data 2), efflux pump determinants are predominant, while antibiotic target replacement contributes to the least extent to the resistome. For a total of 15 antibiotic classes at least one ARG was present in all studied metagenomes (Supplementary Data 2). These include aminocoumarin, aminoglycoside, diaminopyrimidine, fluoroquinolone, glycopeptide, glycylcycline, macrolide, peptide, phenicol, tetracycline, triclosan, sulfonamipeptide, sulfonamide, and β-lactam, whereby ARGs for all β-lactam subclasses (penam, penem, monobactam, cephamycin, cephalosporin and carbapenem) were present in all metagenomes.

Subsequently, the alpha and beta diversity were assessed to compare the richness and structural composition of bacterial communities and ARGs in relation to location and microenvironment (*Sphagnum* vs. non-*Sphagnum*) (Supplementary Tables 3 and 4). Bacterial richness was significantly higher in samples from Austria in comparison to samples from Sweden (*p* < 0.05; Supplementary Table 3). When all resistomes were analysed together, there was no significant difference in the richness between samples from Austria and Sweden (*p* > 0.05; Supplementary Table 3). Moreover, linear regression analysis did not show any correlation between bacterial community structures and resistome richness (*p* > 0.05; Supplementary Table 5). In general, geographic location (Austria vs. Sweden) and microenvironment (*Sphagnum* vs. non-*Sphagnum*) were shown to significantly affect bacterial communities and resistome composition (*p* < 0.05; Supplementary Table 4). Mantel test indicated a significant correlation between bacterial community and resistome composition (*p* < 0.05; Supplementary Table 6). Overall, the data showed that bacterial community composition is the primary determinant of the ARG composition in *Sphagnum*.

In order to compare the short read-based approach with a long read-based analysis and to corroborate the CARD assignment, MAGs (*n* = 298) were reconstructed from metagenomic data and aligned against CARD followed by alignment of the curated hits against the eggNOG database. Within the protein sequences identified as ARGs by CARD, 41% were assigned as the same ARG by eggNOG and 40% as an equivalent ARG (e.g., mdtB - mdtC) or another ARG from the same resistance mechanism (e.g., mdtA – MuxA) (Supplementary Data 2). Similar to the short read-based analysis, efflux pumps were the most abundant mechanisms. ARGs from three efflux pump families were identified: ATP-binding cassette antibiotic efflux pumps (ABC: acrC, B, D), major facilitator superfamily antibiotic efflux pumps (MFS: ermB), and resistance-nodulation-cell division antibiotic efflux pumps (RND: mexE, F) (Supplementary Data 2).

**Fig. 4:**
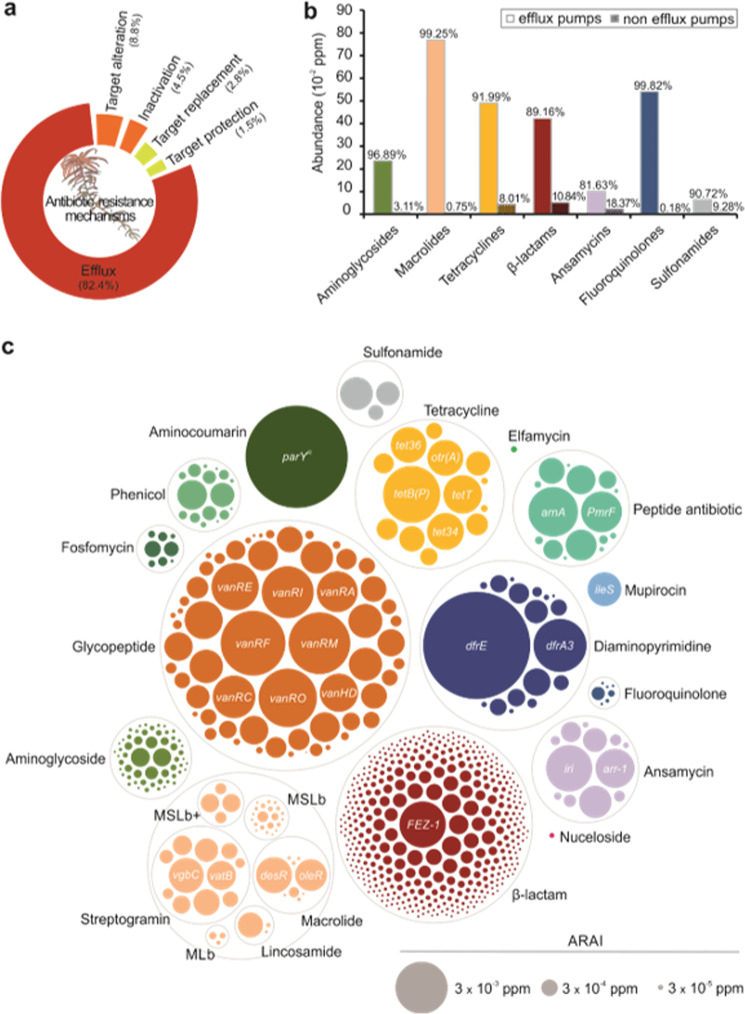
The *S. magellanicum* metagenome comprises a highly versatile resistome. The Illumina generated 41.8 Gbps moss metagenome from the Pirker Waldhochmoor, Austria (N46°37′38.66″, E14°26′5.66″), was aligned against the CARD sequences. **a** The five major resistance mechanisms presented by their relative abundance within the moss resistome. **b** For a selected group of antibiotic classes, the extent of efflux pump mediated and non-efflux pump mediated resistance is compared. Abundance within the metagenome is given in absolute numbers by the ARAI in ppm (≙reads per million reads), while the abundance within antibiotic classes is given as proportion in percent. **c** All detected non-efflux pump related ARGs grouped according to antibiotic classes. Each bubble represents one determinant with absolute abundance within the metagenome reflected by bubble size. The most abundant determinants are labelled with the gene names. MLb macrolide and lincosamide. MSLb, MLb and streptogramin. MSLb+, MSLb and oxazolidione, pleuromutilin and phenicol. CARD Comprehensive Antibiotic Resistance Database, ARAI Antibiotic Resistance Abundance Index.

The highest abundance and diversity among all studied datasets were found in the Austrian *S. magellanicum* metagenome from the Pirker Waldhochmoor (PW) (Fig. 3, Supplementary Data 2). Especially, antibiotic inactivating ARGs were highly versatile (Supplementary Data 2). Due to the high diversity and abundance, the 41.8 Gbps *S. magellanicum* (PW) metagenome was chosen for a more comprehensive analysis. Of its metagenomic reads 0.14% were assigned to 887 ARGs with a collective ARAI [31] of 2.53 ppm. Antibiotic target protection included 19 ARGs and 0.03 ppm (1.3%), antibiotic target replacement 26 ARGs and 0.07 ppm (2.8%), antibiotic target alteration 107 ARGs and 0.23 ppm (9.2%), antibiotic inactivation 512 ARGs and 0.11 ppm (4.6%) and efflux-mediated resistance 220 ARGs, with an extraordinarily high share of collectively 2.07 ppm (82.1%) (Fig. 4a, Supplementary Data 2). Together, the detected resistance determinants span 29 different drug classes.

To understand the strong contribution of efflux pumps towards resistance in more detail, we evaluated the extent of this resistance mechanism against antibiotics at class level (Fig. 4b). Using the *S. magellanicum* (PW) resistome the focus was, thereby, restricted to the antibiotic classes used during the screening of the culture collections. Glycopeptides were omitted as these act on the outer cell wall [9]. Efflux pumps, which export multiple antibiotics, were included in the abundance of each of the respective antibiotic classes. Although to a varying degree between 80% to almost 100%, efflux pumps constituted the most abundant resistance mechanism for all studied classes. Efflux-mediated resistance is more prevalent for macrolides, tetracyclines, β-lactams and fluoroquinolones than for aminoglycosides, rifamycins and sulphonamides based on the determined ARAI (Fig. 4b).

Next, the detected resistance determinants were grouped according to their antibiotic class to compare their distribution and abundance in *S. magellanicum* (PW). However, efflux pumps were excluded entirely in this analysis as they often confer resistance to multiple antibiotics. The overall target spectrum of the detected 667 non-efflux pump determinants spans 20 antibiotic classes including synthetic antibiotics such as diaminopyrimidines, fluoroquinolones and sulphonamides and many classes ranked as critically important for human medicine [62] like aminoglycosides, glycopeptides and β-lactams (Fig. 4c, Supplementary Data 2). These results show a high degree of genetic diversity and an even distribution of the detected ARGs as expected, ranging from 8.3 × 10^−6^ to 1.5 × 10^−2^ ppm (Fig. 4c, Supplementary Data 2). Altogether, the data highlight the predominance of glycopeptide and β-lactam resistance determinants in the studied resistome; both in terms of abundance and versatility with 60 and 403 ARGs and 0.14 ppm (32.6%) and 0.05 ppm (11.6%), respectively.

**Fig. 5:**
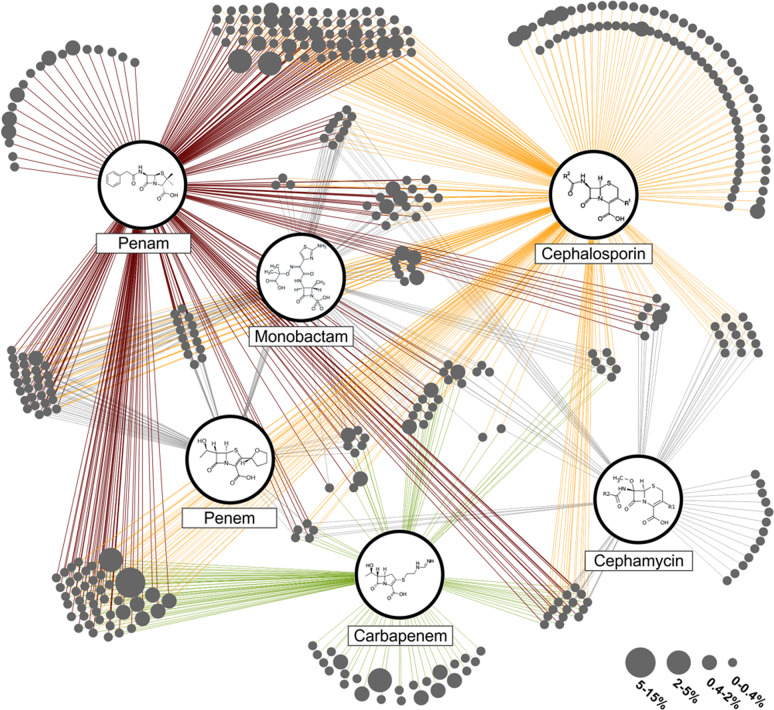
Substrate spectrum of detected β-lactamases. All assigned β-lactamases of the *S. magellanicum* from the Pirker Waldhochmoor, Austria, are displayed. The single β lactamases represented as bubbles (in dark grey) were grouped into clusters based on their reported substrate spectrum. Enzymes with the same substrate spectrum form one cluster. Connecting lines from the clusters to the β-lactam classes display the substrate specificity. Bubble size relates to the relative abundance of single enzymes within the whole β-lactamase pool. The three most abundant classes are penam (red), cephalosporin (orange) and carbapenem (green).

The high β-lactamase diversity in the *S. magellanicum* (PW) resistome covers every β-lactam class including extended spectrum as well as metallo β-lactamases of environmental but also clinical origin, such as GIM-2, SHV-16 and TEM-102 (Supplementary Data 2). Due to the relevance of extended-spectrum and metallo β-lactamases, which pose a problem for the still widely administrated β-lactams, a network analysis was conducted to assess the target spectrum of the 398 assigned β-lactamases (Fig. 5). All six β-lactam classes, penams, penems, monobactams, cephalosporins, cephamycins and carbapenems, are represented in the constructed network. The majority of determinants (67.6%) cluster in groups acting on more than one β-lactam class. These clusters often connect to three, four and five β-lactam classes comprising 22.1%, 13.3% and 6.5% of the detected determinants, respectively. Overall, the determinants connect most frequently to cephalosporins and penams. However, connections to carbapenems, drugs of last resort, are also highly represented in the network analysis.

### Identification of the novel class A β-lactamase Mm3 from the *Sphagnum* metagenome

To assess the potential of the *Sphagnum* resistome to harbour novel resistance genes, a functional metagenomics approach was pursued. The screening of a 3.6 Gbps fosmid library against nine different antibiotics identified three unique resistant metagenomic clones (*E. coli* EPI300 pCC2FOS-Mm1, Mm2 and Mm3); all three conferring resistance against ampicillin. The initially determined MICs for ampicillin were 64 µg ml^−1^ for clones Mm1 and Mm2, and >512 µg ml^−1^ for Mm3, as compared to 32 µg ml^−1^ for the control strain (Supplementary Table 7). The clone *E. coli* EPI300 pCC2FOS-Mm3, exhibiting the highest MIC for ampicillin, was chosen for de novo sequencing. This revealed a novel β-lactamase gene encoding a 304 amino acid protein with an estimated weight of 32.8 kDa to be present on the 40.7 kb DNA insert. The gene was designated *blaMm3* (β-lactamase from Moss metagenome clone 3).

To see if the novel Mm3 β-lactamase is present in the studied metagenomes and to compare it to known β-lactamases, a blastX search against all studied metagenomes and in general against the non-redundant protein sequence database (NCBI) was performed. This resulted in sequence identities of no more than 84%, with only one exception: one metagenomic read with 97% sequence identity at the amino acid level (99% at DNA level) originating from the same microbiome as the Mm3 β-lactamase (Pirker Waldhochmoor, Austria) (Supplementary Fig. 2).

**Fig. 6:**
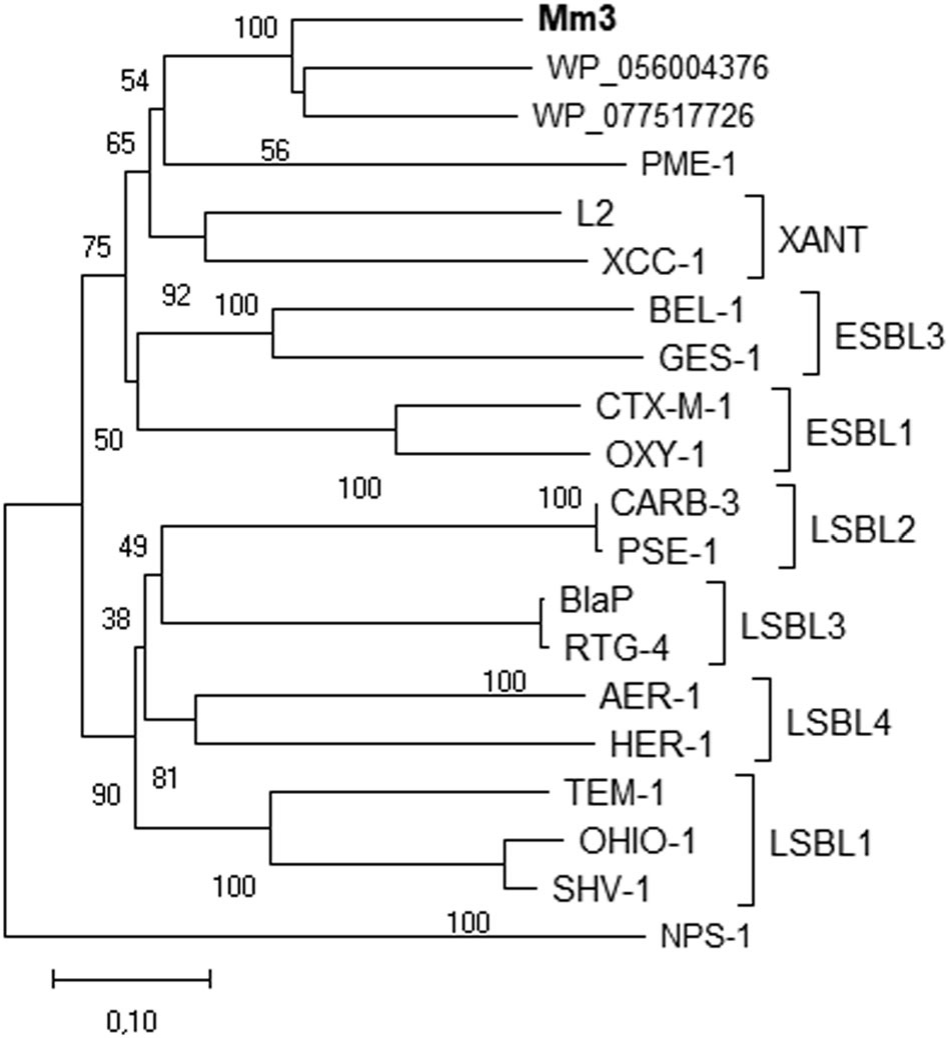
Phylogenetic relationship of Mm3 and other class A β-lactamases. The evolutionary analysis of aligned amino acid sequences was conducted using the neighbour-joining method. Bootstrap values are shown next to the branches. The scale bar indicates the number of amino acid differences per sequence. The reference sequences are: *Frateuria* sp. Soil773 (WP_056004376), *Rhodanobacter* sp. C03 (WP_077517726), PME-1 (*Pseudomonas aeruginosa*, E9N9H5), L2 (*Stenotrophomonas maltophilia*, P96465), XCC-1 (*Xanthomonas campestris* pv. *campestris*, O87643), BEL-1 (*Pseudomonas aeruginosa*, Q3SAW3), GES-1 (*Klebsiella pneumoniae*, Q9KJY7), CTX-M-1 (*Escherichia coli*, P28585), OXY-1 (*Klebsiella oxytoca*, P22391), CARB-3 (*Pseudomonas aeruginosa*, P37322), PSE-1 (*Pseudomonas aeruginosa*, Q03170), AER-1 (*Aeromonas hydrophila*, Q44056), HER-1 (*Escherichia hermannii*, Q93FN7), BlaP (*Proteus mirabilis*, P30897), RTG-4 (*Acinetobacter baumannii*, ACJ61335), TEM-1 (*Shigella flexneri*, AAC97980), OHIO-1 (*Enterobacter cloacae*, P18251), SHV-1 (*Klebsiella pneumoniae*, P0AD64). The tree was rooted with NPS-1, a class D β-lactamase from *Pseudomonas aeruginosa* (AAK1479).

Subsequently, the phylogenetic relation was inferred to allow classification of the novel β-lactamase gene *blaMm3*. Based on the generated phylogeny, *Mm3* shares the highest sequence similarity with two annotated but not yet characterised β-lactamases from *Rhodanobacter* sp. (70.6%) and *Frateuria* sp. (66.8%). Both species belong to the family of *Rhodanobacteraceae* and order of *Xanthomonadales*. According to the updated classification by Philippon et al. [63], elucidation of the evolutionary relatedness of Mm3 with reference sequences from characterised β-lactamases, showed that the novel β-lactamase clustered, together with the next neighbour sequences from *Rhodanobacter* sp. and *Frateuria* sp., in closer proximity to members of the so called *Xanthomonas* (XANT) group (Fig. 6). The XANT group contains β-lactamases from *Xanthomonas* spp., *Stenotrophomonas maltophilia* and *Pseudomonas aeruginosa*. Other clusters in the phylogenetic tree include members showing a lower degree of similarity (33–44% identity), like those belonging to the limited-spectrum (LSBL1 to 4) and extended-spectrum β-lactamases (ESBL1 and 3). Members of the LSBL2 and 3 clusters have been described as true carbenicillinases, while enzymes from the ESBL group hydrolyse cephalosporins like cefotaxime additionally to penicillins. In accordance with the phylogenetic analysis, the amino acid sequence of Mm3 harbours characteristic class A Ambler motifs [64] as follows: 70SerThrPheLys (SxxK motif), 130SerAspAsn (SDN motif), 234LysThrGly (KTG motif), Glu166 and 166GluProGluLeuAsn (ExxLN motif).

When the substrate spectrum of the novel β-lactamase was determined, clone Mm3 showed no to little resistance to the tested cephalosporin concentrations with MICs of <0.5, 64 and 8 µg ml^−1^ for cefatoxime, cephalexin and cefalothin, respectively (Supplementary Table 7). This was similar to the control strain. On the contrary, Mm3 displayed a distinctly higher resistance against the penam antibiotics ampicillin (>512 µg ml^−1^) and carbenicillin (>1024 µg ml^−1^).

Finally, the novel β-lactamase Mm3 was biochemically characterised. After recombinant expression of the novel β-lactamase Mm3, the soluble and active N-terminally His-tagged enzyme (as confirmed by SDS-PAGE analysis and testing cell-free lysates on nitrocefin disks) (data not shown), was purified by affinity chromatography to a purity of 90% as estimated by SDS-PAGE. Two prominent bands with a molecular weight of around 32 and 35 kDa were visible (Supplementary Fig. 3). LC-MS/MS analysis of both bands determined each of the respective proteins to comprise the right β-lactamase amino acid sequence (data not shown). However, the 35 kDa protein contained the N-terminal His-Tag while the lower weight protein did not (32 kDa), probably as a result from proteolytic activity during purification or SDS-PAGE analysis. The kinetic analysis revealed a higher affinity of Mm3 for ampicillin (V_max_ = 179.2 ± 6.1 U mg^−1^, K_M_ = 270.8 ± 16.4 µM; fitted with the Hill equation [n_H_ of 2.36]) as compared to carbenicillin (V_max_ = 264.6 ± 8.6 U mg^−1^, K_M_ = 399.85 ± 42.69 µM; fitted with the Michaelis-Menten equation) (Supplementary Fig. 4).

## Discussion

Our multi-faceted analysis of different bog microbiomes across Europe uncovered a highly versatile resistome present in the evolutionary old and long-term stable bog ecosystem. The obtained results underline the natural, strong resilience of *Sphagnum-*associated bacteria against antibiotics. Given the highly adapted plant-associated lifestyle, the strong microbial competition and the vast pool of microbial and plant-produced secondary metabolites [12], the *Sphagnum* microbiota has developed general and also specific antimicrobial resistance mechanisms that naturally equip them against antibiotics. (Semi)synthetic drugs were not exempt from this as demonstrated in the present study. Contrary to our initial expectation, a predominance of resistances against (semi)synthetic antibiotics was observed in the culture collections, despite their pristine origin [10]. Although likely stemming to a large part from efflux pumps as indicated by the in silico analysis, the ability to combat these compounds may not exclusively result from extrusion. For instance, resistance of environmental bacteria against synthetics has been tied to high sequence variations in target genes [65].

As outlined by the comparative analysis, the isolated bacteria cluster independent of geographic location and plant species into separate groups depending on their resistance profile. Based on this we hypothesise that a shared core resistome might be present in *Sphagnum*-dominated bogs independent of geographic location. As *Sphagnum*-bogs share a geographically independent core microbiome [13, 16] and given the association of certain ARGs with certain taxa [66–69], these ecosystems may not only possess a core microbiome, but also a core resistome. However, this hypothesis remains to be verified by targeted analyses with additional data from different geographical origin. The occurrence of a shared core resistome across these old ecosystems would imply that a high resistome stability is likely facilitated through the ecological mechanisms and the environmental conditions therein. *Sphagnum* mosses are known to transmit specific microbiota to the next generation and maintain them throughout their life cycle [14, 70]. Consequently, species specific ARGs will be transmitted. In addition, *Sphagnum*-dominated peatbogs miss antimicrobial selection pressure to drive resistome shifts. Resistome stability is, thereby, a phenomenon not exclusive to pristine plants, as just recently described for resistomes associated with the human oral cavity and stool [69].

This finding is reinforced by the results of the in silico analysis, which displayed a geographically independent structure of resistance mechanisms. The bog resistome further exhibits a high versatility and evenness of ARGs and, thus, reflects the typical characteristics of the bog microbiome (stable and high microbial diversity, geographic independently shared core microbiome). Given the high stringency applied to our analysis, the observed coverage on the functional and chemical level with a total of 943 ARGs covering 29 antibiotic classes is staggering. This includes *aph, sul* and other indicator genes, clinically relevant and environmentally prevalent ARGs proposed by Berendonk et al. as markers to survey the antibiotic resistance status in different environments [8]. Due to the pristine nature of the studied peat bogs the identified indicator-ARGs are natural constituents of the studied resistome and should not be regarded as pollutants. Functional predictions using whole genome sequences are vulnerable to false-positive results unless complementary biochemical or phenotypical assessment are performed to validate the result [71]. Nevertheless, such approaches are valuable because they are not restricted to cultivable microorganisms and thus can complement classical screenings to improve surveillance of antibiotic resistance within microbial communities [72–74].

A potential key for the observed vast diversity of ARGs resides in the presence of a great repertoire of efflux pumps (63–98% abundance). These multi-protein complexes are ubiquitous in prokaryotes whereby many species possess several different efflux pumps [3, 9]; especially soil and plant bacteria showcase some of the largest numbers of efflux pumps per genome [75]. Considered an evolutionary ancient and general resistance mechanism they confer resistance against several antibiotics, toxic molecules like heavy metals, solvents, and plant-produced antimicrobials [75]. Their physiological role further includes the extrusion of endogenous toxic compounds, cell-cell communication through the transport of quorum sensing molecules or maintenance of the intracellular pH [75]. Consequently, efflux pumps play an important role for environmental adaption and interaction with host plants – colonisation and *in planta* survival – and may be a missing link in understanding resistomes in natural environments (especially those under little to no anthropogenic influence). The *Sphagnum* microbiota is characterised by a high taxonomic diversity which inherently is a driving force for a vast diversity of microbial chemicals and molecules in addition to the array of plant produced compounds. In addition, the rich *Sphagnum*-microbiota inhabit these mosses in close proximity to one another as highlighted by the confocal scanning laser microscopy. Within this densely populated, highly complex community, efflux pumps ensure co-existence and thereby facilitate species richness and concomitantly a diverse pool of ARGs as present in the moss resistome. In doing so, they contribute to the great resilience found within the peat bog ecosystem.

Due to the shared resistome characteristics of bog microbiomes and the great resilience present therein, it is not surprising, that a high level of multi-resistance was encountered in all three countries during the culture-dependent analysis. It was previously already proposed that most microorganisms might naturally be multi-resistant [9]. Yet, an in-depth analysis to assess entire plant microbiomes for multi-resistance has not been conducted. The results reinforce Wright´s hypothesis and provide further proof for it in the context of the microbiota associated with pristine and evolutionary old moss species. The observation that abundance of multi-resistance is associated to geographic location has to be interpreted carefully. This tendency more likely stems from the characteristics of the culture collections. The German and Norwegian collections were generated upon pre-selection of bacteria with antifungal activity [13, 17], while no pre-selection was made for the Austrian culture collection. Based on this observation we hypothesise that antagonistic bacteria likely naturally possess a higher proportion of resistances, which would need to be evaluated in future studies.

All sequenced multi-resistant isolates were assigned to *Proteobacteria;* the majority belonging to *Burkholderiales* and *Enterobacteriales*, which are typical and dominant orders within the bacterial community of *Sphagnum* mosses [12, 14]. It is therefore not surprising that erythromycin and vancomycin accounted as the two most important determinants to discriminate between low and high resistance profiles in this dataset. Gram-negative bacteria often possess intrinsic resistance against both these antibiotics [9, 76, 77]. *Serratia* spp*., Pseudomonas* spp*., Paraburkholderia* spp., and *Rouxiella* sp. were isolated from this habitat before [17, 78, 79]. For *Pandoraea* spp. an association with *Sphagnum* was not described so far. Interestingly, this bacterium has been mostly isolated from the sputum of cystic fibrosis patients [80] and is considered as emerging opportunistic pathogen [81]. Clinically isolated *Pandoraea* spp. are known to be highly resistant, including resistances to last defence antibiotics of the carbapenem class [81, 82]. Our results show that *Pandoraea* spp. naturally possess a high level of resistance and they might easily transit to clinical environments as they are well equipped with ARGs. Accordingly, assessment of the environmental resistome in a given habitat can be used to better understand or even predict emerging opportunistic and multi-resistant pathogens. As for *Serratia* spp. and *Pseudomonas* spp., these are common plant-associated bacteria, which also exhibit various antibiotic resistances and opportunistic traits, and are commonly associated to human infections, in particular *Serratia marcescens* and *Pseudomonas aeruginosa* [83, 84]. Less is known to this end for the identified *Paraburkholderia* and *Rouxiella* species, especially for *Paraburkholderia phytofirmans*, which is a promising plant growth promoting agent [85]. Interestingly, plants in general and *Sphagnum* in particular constitute reservoirs for plant growth promoting bacteria with antifungal and antibacterial activity [86], while simultaneously hosting species known as opportunistic human pathogens [17, 87] as well as emerging nosocomial pathogens, e.g., *Pandoraea*.

Our data highlight that the plant microbiome naturally comprises a versatile, intrinsic resistome. This is reinforced by the identification of a novel class A β-lactamase. Notably, Mm3 shares low sequence similarity to other annotated β-lactamases. This is a common observation for metagenome-derived ARGs [76] and in case of the prospected habitat can be explained by the fact that most moss-associated microorganisms are not yet cultivable. Hence, not much is known about the origin and genetic content of *Sphagnum* microbiota [12]. The metagenome derived β-lactamase Mm3 is phylogenetically closest related to β-lactamases of the XANT group and the uncharacterised β-lactamases from the environmental isolates *Rhodanobacter* (soil) and *Frauteuria* (rhizosphere), both belonging to the order of *Xanthomonadales* [88, 89]. *Xanthomonadales spp*. constitute common colonisers of *Sphagnum* mosses [16, 70]. Interestingly, many of the other β-lactamase sequences clustering in close proximity were isolated from well-known nosocomial human pathogens such as *S. maltophilia*, *P. aeruginosa* or *Klebsiella pneumoniae* [81]. The relatedness of *bla*Mm3 to these genes is not surprising, since β-lactamases account as evolutionary old enzymes and are widely spread in nature [9]. The latter was clearly confirmed in the network analysis, displaying high abundance and an extraordinary diverse substrate range of the in silico detected β-lactamases. The isolated β-lactamase Mm3 showed a higher affinity for penam antibiotics, but no activity for the tested cephalosporins, exhibiting in this case a narrow substrate spectrum. With K_M_ values around 270–400 µM for the penam antibiotics, the activity of the new Mm3 is not outstanding and surpassed by the ones reported for β-lactamases from many facultative human pathogens, e.g., the plasmid-encoded MIR-1, CMY-1 or ACT-1 from *E. coli* (0.16 to 2.2 µM, ampicillin) [90–92]. This raises the question whether antibiotic-inactivating enzymes in natural environments possess a rather limited activity as compared to the clinical settings.

Since *Sphagnum* mosses are rootless plants that do not have soil contact within peat bogs and the majority of their associated bacteria is transferred from the sporophyte to the gametophyte [14], the elucidated resistome can be mostly regarded as inherent to the *Sphagnum* microbiome. As such, it addresses the need to understand the extent to which the plant resistome is inherent or recruited from soil, which represents still an unanswered question [5]. As emphasised earlier, we, further, expect that some of the inherent *Sphagnum*-associated ARGs will be vertically transmitted with the core microbiome from the gametophyte to the sporophyte and vice versa [14]. Consequently, the vertical transmission of ARGs through passing on their bacterial carriers adds to the development and maintenance of the core resistome, which based on our analysis features ARGs of four resistance mechanisms (efflux, target inactivation, alteration and protection; target replacement in one third of the samples) and 15 antibiotic classes. Interestingly, Carr et al. recently noted similar resistome patterns for the human oral cavity and gut [69]. They describe a similar distribution of resistance mechanisms and highlight the occurrence of cephamycin, fluoroquinolone, macrolide and tetracycline ARGs in all their samples, which was observed for the bog ecosystems as well. Although not found in all samples, aminoglycosides, glycopeptides, peptides, phenicol and sulphonamide constitute further antibiotic classes commonly found in bog metagenomes and by Carr and colleagues in human metagenomes. This also includes many shared ARGs such as *acrD*, *patA*, *mdtF* and *mdtB* and indicates that certain ARGs are native in plant as well as in human microbiomes.

Since ARGs are often associated with specific taxa [67–69, 93], we expected the taxonomically diverse and balanced *Sphagnum-*associated microbiome to comprise a resistome with evenly distributed ARGs at low abundance; a common observation for natural environments [4]. In contrast, environments under anthropogenic influence are characterised by highly abundant ARGs [4, 68], and further correlate with a loss in bacterial diversity and enrichment of opportunistic pathogens [68]. The antimicrobial selective pressure exerted by our life style is without question the driving force for imbalance, leading to a shift in bacterial community composition that ensues the increase of opportunistic pathogens and their associated ARGs. However, potentially overlooked in this context is the ecological concept of *K*- and *r*-selection favouring oligotrophic or copiotrophic taxa, respectively. We recently reported that the phyllosphere of arugula from urban gardening was dominated by *Gammaproteobacteria* and in particular by multi-resistant *Enterobacteriacaea* [6]. This class, which comprises many opportunistic pathogens, tends towards a copiotrophic lifestyle, displaying faster growth and substrate generalisation as compared to the more oligotrophic *Alphaproteobacteria* [94]. The *Sphagnum* microbiota in contrast is dominated by *Alphaproteobacteria*, such as the slow growing *Methylobacteria* [12, 14]. We assume that *K*-selection maintains oligotrophy and stabilises the bacterial community in the nutrient poor, microbial rich ecosystem of *Sphagnum-*dominated bogs. In doing so it represents a driving force in shaping the observed evenness and diversity within the moss resistome. Microbial community management ensuing diverse, stable and beneficially designed microbiomes is foreseen to abate exposure to resistances [68]. We propose that resistance management in form of microbial community management could be achieved through *K*-selection. The advantageous effects of such a strategy have already proven valuable in improving the larvae viability in aquaculture [95].

Based on our complementary screening strategy, the herein presented novel findings deliver a first comprehensive picture of native plant core resistomes across different European bog ecosystems, showing geographically independent patterns and consisting of a highly diverse genetic pool and novel antibiotic resistance genes. As such the results are in line with previous work that highlights the global and natural occurrence of resistances. This substantiates the importance of addressing the knowledge gap about plant and pristine resistomes in future research. These habitats constitute ideal subjects to unravel the ecological principles driving resistome stability and shifts, fundamental for our understanding of the anthropogenic influence on resistance emergence and spread and for the development of resistance management strategies.

## Supplementary information

Supplementray Video 1

Supplemental Material

Supplementary Data 1

Supplementary Data 2
