# Supplementary material for: Plant resistome profiling in evolutionary old bog vegetation provides new clues to understand emergence of multi-resistance
Source: ISME J. 2020 Nov 11;15(3):921–37. doi: 10.1038/s41396-020-00822-9 (PMC8027415; doi:10.1038/s41396-020-00822-9)
Supplement: Supplementary file 2 — Supplemental Material [file 41396_2020_822_MOESM2_ESM.docx]

**Plant resistome profiling in evolutionary old bog vegetation provides new clues to understand emergence of multi-resistance**

**SUPPLEMENTARY MATERIAL**

Melanie Maria Obermeier^1,2^, Wisnu Adi Wicaksono^1^, Julian Taffner^1^, Alessandro Bergna^1,2^, Anja Poehlein^3^, Tomislav Cernava^1^, Stefanie Lindstaedt^4^, Mario Lovric^4^, Christina Andrea Müller Bogotá^1,2^*, Gabriele Berg^1,2^

^1^Institute of Environmental Biotechnology, Graz University of Technology, Petersgasse 12/I, 8010 Graz, Austria

^2^ACIB GmbH, Krenngasse 37/II, 8010 Graz, Austria

^3^Göttingen Genomics Laboratory, Institute for Microbiology and Genetics, Georg-August-University, Grisebachstrasse 8, 37077 Göttingen, Germany

^4^Know-Center GmbH, Research Center for Data-Driven Business & Big Data Analytics, Infeldgasse 13/VI, 8010 Graz, Austria

*Corresponding author:

Christina Andrea Müller Bogotá

Institute of Environmental Biotechnology

Graz University of Technology, Petersgasse 12

8010 Graz, Austria

christina.mueller@tugraz.at

Note, that a detailed table listing all the identified ARGs and their abundances along with information on all analysed datasets can be found in Supplementary Data 2.

**Figures**


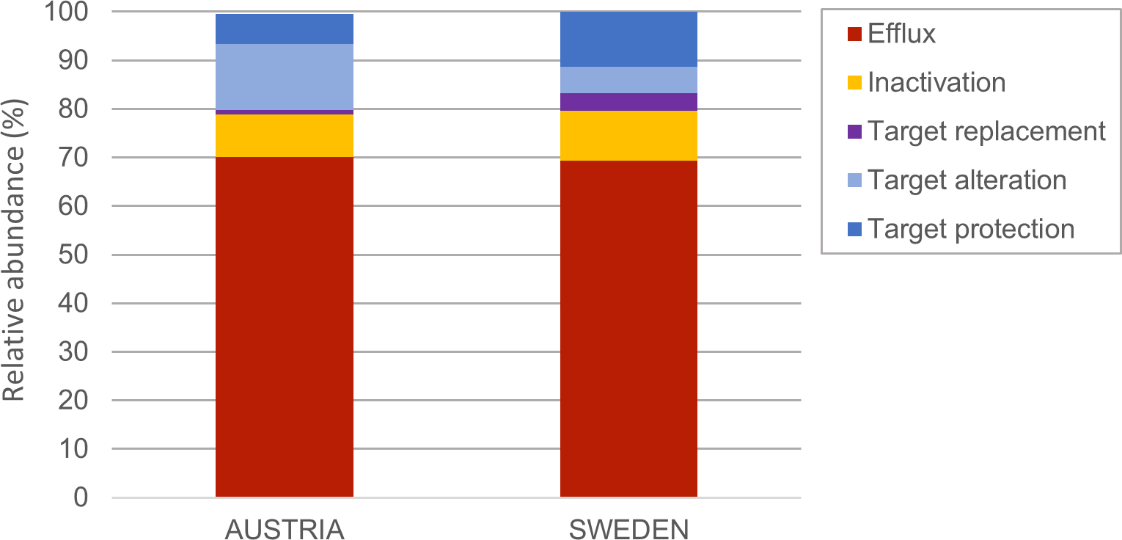


**Supplementary Figure 1: Relative abundance of resistance mechanisms for *Sphagnum-*dominated bogs.** The relative abundance of each resistance mechanism is shown jointly for all Austrian and a Swedish peat bog, displayed as mean value.


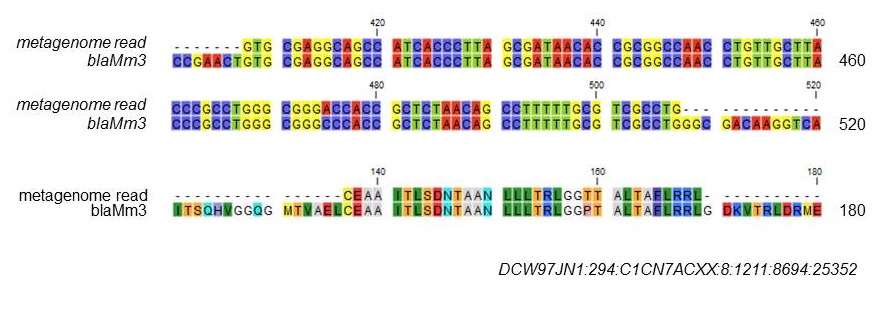


**a**

**b**

**Supplementary Figure 2: Alignment of Mm3 β-lactamase and metagenome read.** Nucleotide (a) and amino acid (b) sequence of the metagenome read DCW97JN1:294:C1CN7ACXX:8:1211:8694:25352 from the Pirker Waldhochmoor (N46°37′38.66″, E14°26′5.66″) microbiome aligning to nucleotide 408-507 and amino acid 137-169 of the Mm3 β-lactamase.


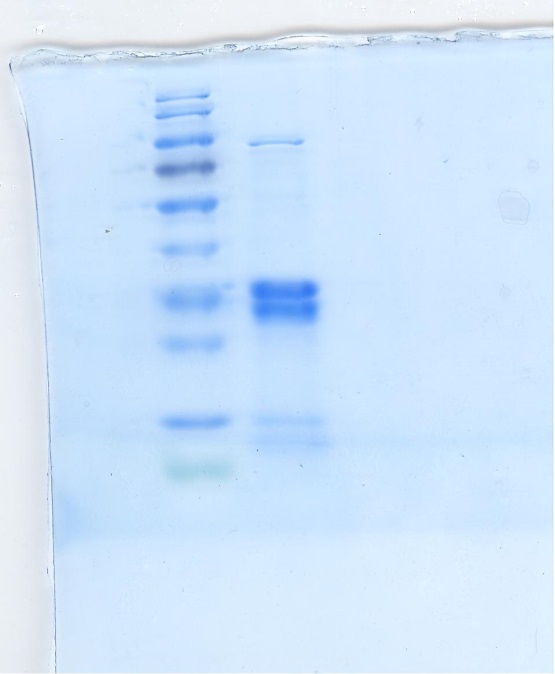

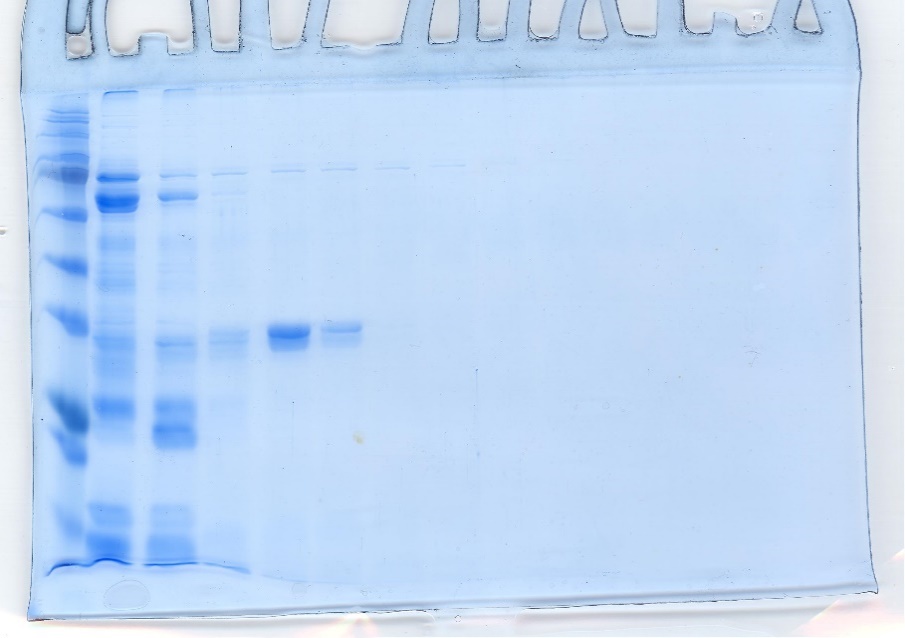


kDa

100

25

22

17

32

46

58

80

kDa

40

35

10

55

70

100

130

25

15

14 15 16 17 18 19

**a**

**b**

**Supplementary Figure 3: Purified β-lactamase Mm3.** The *blaMm3* gene encoding a 304 amino acid long β-lactamase was cloned under an N-terminal His Tag and the tagged protein purified by affinity chromatography. a) Elution fractions no. 14 to 19; no. 17 and 18 were selected for further use. b) SDS-PAGE of the pure enzyme shows two bands, one with the estimated molecular weight of the his-tagged protein of about 35 kDa and a smaller protein band around 32 kDa. As identified by LC-MS/MS analysis the higher molecular weight band contains the β-lactamase still adjunct to the His-Tag, while the lower band stems from a smaller version of the purified β-lactamase which lost the His-Tag. Lower molecular weight proteolytic products of approximately 15 to 17 kDa are visible as well, corresponding to the His-tagged termini of the protein. Protein Ladder: a) Color prestained Protein Standard, Broad Range (New England Biolabs); b) PageRuler Prestained Protein Ladder (Thermo Scientific).

**Supplementary Figure 4: Kinetic characterisation of the β-lactamase Mm3.** The initial hydrolysis of the substrate was followed spectrophotometrically at 235 nm. Points, mean values (n = 2 to 8) for all measurements, except for 2.5 mM carbenicillin (n=1); error bars, standard deviations. Data for ampicillin (a) and carbenicillin (b) were fitted according to the Hill or Michaelis-Menten equation, respectively.


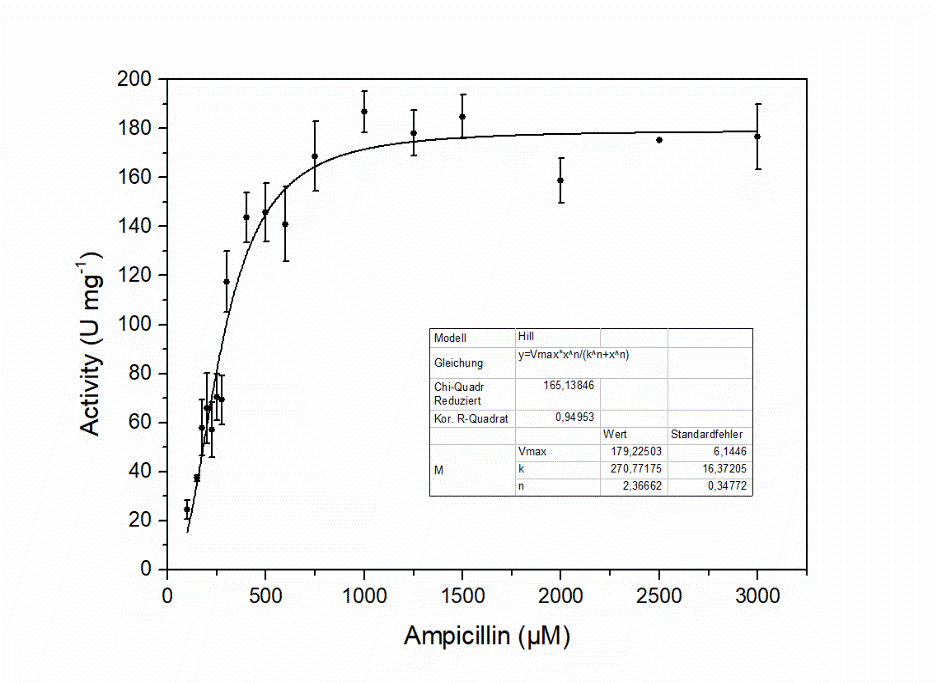

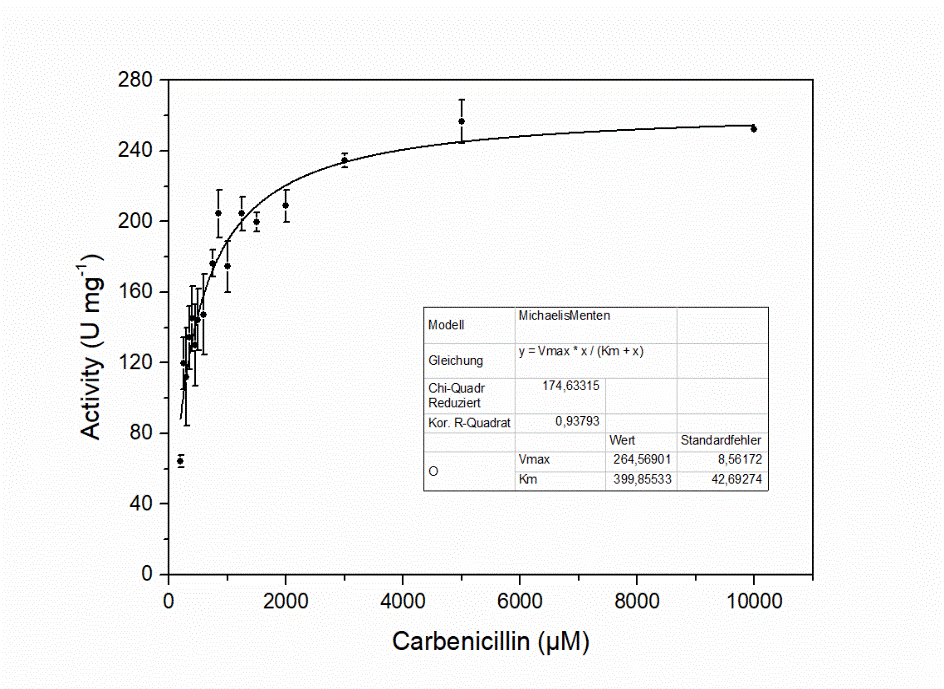


**a b**

**Tables**

**Supplementary Table 1: Antibiotic concentrations for resistance screenings used in this study.**

| **Antibiotic** | **Antibiotic class** | **Manufacturer** | **Spectrum** | **Concentration**  **[µg ml ^-1^] used for** | |
| --- | --- | --- | --- | --- | --- |
|  |  |  |  | **Isolates** | **Metagenomic clones** |
| Ampicillin | β-Lactam | Roth, Germany | Gram +/- | 10 | 50 |
| Ciprofloxacin | Fluoroquinolone | Sigma-Aldrich, Missouri, USA | Gram +/- | 5 | 1 |
| Erythromycin | Macrolide | Roth, Germany | Gram +/- | 15 | 150 |
| Gentamycin | Aminoglycoside | Roth, Germany | Gram +/- | 10 | 10 |
| Kanamycin sulfate |  | Roth, Germany | Gram +/- | 30 | 20 |
| Nalidixic acid | Quinolone | Merck, Germany | Gram +/- | 30 | 15 |
| Tetracycline | Tetracycline | Merck, Germany | Gram +/- | 30 | 4 |
| Rifampicin | Ansamycin | Duchefa Biochemie, Netherlands | Gram +/- | 5 | 20 |
| Sulfadiazine | Sulfonamide | Sigma-Aldrich, Missouri, USA | Gram +/- | 300 | ≤ 2250 |
| Vancomycin | Glycopeptide | Sigma-Aldrich, Missouri, USA | Gram + | 30 | 1000 |

**Supplementary Table 2: List of manually curated resistance determinants exemplified by *S. magellanicum* (PW).** Abundance is given in number of assigned reads before and after curation and after normalisation of the curated abundance as ARAI*. a) Genes which represent antibiotic targets, for which point mutations are known to confer resistance. Assigned reads which align to the mutation area were filtered and only those containing the point mutation were retained in the dataset. b) Genes and their assigned Antibiotic Resistance Ontologies (AROs) to which two or more gene sequences are attributed to. To avoid multiple representations of such AROs, hits of homolog genes were merged. c) Genes sourced out due to high similarity to common and widely spread genes, which are not directly related to antibiotic resistance.

|  | Gene  (ARO) | Abundance before curation | A) Mutation  (No. of reads aligning to mutation area)  B) Accession numbers of homologous genes | Abundance  after curation | ARAI* (ppm) after normalisation of curated reads |
| --- | --- | --- | --- | --- | --- |
| 1. Curated for mutations | *Chlamydia trachomatis murA*  (ARO:3003785) | 194 | C119D  (0) | 0 | 0 |
|  | *Mycobacterium tuberculosis murA* (ARO:3003784) | 1058 | C117D  (309) | 22 | 0.00030495 |
|  | *Streptomyces cinnamoneus EF-Tu* (ARO:3003359) | 13978 | A379T  (1039) | 9 | 0.00013135 |
| 1. Curated for   gene redundancy | *arnA*  (ARO:3002985) | 361 | NP_252244 | 1007 | 0.00884032 |
|  |  | 646 | AAC75315.1 |  |  |
|  | *cat*  (ARO:3002670) | 114 | AAA22081.1 | 118 | 0.00317999 |
|  |  | 2 | AAA23018.1 |  |  |
|  |  | 1 | BAC11901.1 |  |  |
|  |  | 1 | AAB23649.1 |  |  |
|  | *catIII*  (ARO:3002685) | 1 | CAB75601.1 | 2 | 5.4404E-05 |
|  |  | 1 | CAA30695.1 |  |  |
|  | *ANT(6)-Ib*  (ARO:3002629) | 1 | CBH51824.1 | 2 | 4.0236E-05 |
|  |  | 1 | AIJ27543.1 |  |  |
| 1. Other | *mfd* (ARO:3003844) | 8097 | ** | 0 | 0 |
|  | *NmcR* (ARO:3003665) | 442 | *** | 0 | 0 |

*ARAI (antibiotic resistance abundance index): number of reads assigned to an antibiotic resistance gene per total number of reads and respective gene length in ppm (≙reads per million reads)^1^.

** *Mfd* influences the spontaneous mutation rate that can give rise to ciprofloxacin resistances in *Campylobacter jejuni*^2^. It is, however, a wide-spread protein involved in DNA repair and by itself not directly related in antibiotic resistance^3^.

*** *NmcR* regulates the *NmcA* β-lactamase, to which one metagenomic read was assigned resulting in an abundance of 1,98E-05 ppm (Supplementary Data 2). However, *NmcR* is a homolog of the widely conserved *lysR* regulators^4^.

**Supplementary Table 3:** Summary of the results from statistical analyses. The effects of geographical location (Austria vs. Sweden) and microenvironment (*Sphagnum* vs. non-*Sphagnum*) on bacterial communities and resistome richness (alpha diversity) are shown.

| Indicator | All datasets | All datasets | *Sphagnum* only dataset | Austria only dataset |
| --- | --- | --- | --- | --- |
|  | *Sphagnum* vs. non-*Sphagnum* | Sweden vs. Austria | Sweden vs. Austria | *Sphagnum* vs. non-*Sphagnum* |
|  | p value | p value | p value | p value |
| Bacterial richness^#^ |  |  |  |  |
| rplC | 0.34 | 0.026 | 0.034 | 0.817 |
| S5 | 0.491 | 0.031 | 0.05 | 0.537 |
|  |  |  |  |  |
| Resistome richness^$^ |  |  |  |  |
| All resistome | 0.101 | 0.201 | 1 | 0.355 |
| Efflux pump | 0.125 | 0.459 | 0.724 | 0.217 |
| Antibiotic inactivation | 0.017 | 0.158 | 1 | 0.064 |
| Antibiotic target alteration | 0.03 | 0.026 | 0.289 | 0.28 |

^#^Bacterial richness was calculated based on the Shannon diversity index and analysed using Kruskal Wallis test

^$^Resistome richness was calculated based on Shannon diversity index and analysed using Kruskal Wallis test. The subset of ARGs that have belong to a different mechanism than efflux pumps, antibiotic inactivation and antibiotic target alteration were analysed separately.

**Supplementary Table 4:** Summary of results from statistical analyses testing effects of geographical location (Austria vs. Sweden) and microenvironment (*Sphagnum* vs. non-*Sphagnum*) on bacterial communities and resistome composition (beta diversity).

| Indicator | All dataset | | All dataset | | *Sphagnum* only dataset | | Austria only dataset | |
| --- | --- | --- | --- | --- | --- | --- | --- | --- |
|  | *Sphagnum* vs. non-*Sphagnum* | | Sweden vs. Austria | | Sweden vs. Austria | | *Sphagnum* vs. non-*Sphagnum* | |
|  | p value | R^2^ | p value | R^2^ | p value | R^2^ | p value | R^2^ |
| Bacterial composition^#^ |  |  |  |  |  |  |  |  |
| rplC | 0.074 | 0.054 | 0.134 | 0.001 | 0.310 | 0.023 | 0.093 | 0.033 |
| S5 | 0.074 | 0.05 | 0.134 | 0.001 | 0.302 | 0.030 | 0.093 | 0.050 |
|  |  |  |  |  |  |  |  |  |
| Resistome composition^$^ |  |  |  |  |  |  |  |  |
| All resistome | 0.123 | 0.008 | 0.161 | 0.001 | 0.348 | 0.027 | 0.147 | 0.014 |
| Efflux pump | 0.121 | 0.020 | 0.169 | 0.004 | 0.354 | 0.031 | 0.146 | 0.044 |
| Antibiotic inactivation | 0.078 | 0.140 | 0.201 | 0.002 | 0.363 | 0.028 | 0.099 | 0.245 |
| Antibiotic target alteration | 0.110 | 0.091 | 0.127 | 0.061 | 0.312 | 0.058 | 0.128 | 0.158 |

^#^Bacterial composition differences were assessed using Adonis.

^$^Resistome composition analysis was performed using Adonis. A subset of ARGs that are based on different mechanisms than efflux pumps, antibiotic inactivation and antibiotic target alteration were analysed separately.

**Supplementary Table 5:** Summary of the results obtained with linear regression analyses.

|  | Resistome | Efflux pump | Antibiotic inactivation | Antibiotic target alteration |
| --- | --- | --- | --- | --- |
|  | p value | p value | p value | p value |
| rplC | 0.949 | 0.793 | 0.376 | 0.925 |
| S5 | 0.782 | 0.941 | 0.348 | 0.729 |

**Supplementary Table 6:** Summary of results from the Mantel test analyses.

|  | Resistome | | Efflux pump | | Antibiotic inactivation | | Antibiotic target alteration | |
| --- | --- | --- | --- | --- | --- | --- | --- | --- |
|  | p value | r | p value | r | p value | r | p value | r |
| rplC | 0.002 | 0.291 | 0.003 | 0.253 | 0.004 | 0.428 | 0.023 | 0.191 |
| S5 | 0.003 | 0.319 | 0.001 | 0.274 | 0.005 | 0.432 | 0.017 | 0.202 |

**Supplementary Table 7: Minimal inhibitory concentrations of β-lactam resistant metagenomic clones.** Minimal inhibitory concentration (μg ml^-1^) of penam and cephalosporin antibiotics for the metagenomic clones *E. coli* EPI300 pCC2FOS-Mm1, Mm2 and Mm3 and the empty vector control *E. coli* EPI300 pCC2FOS (X). Mean values, n = 3.

| Clone | Ampicillin | Carbenicillin | Cefotaxime | Cefalothin | Cephalexin |
| --- | --- | --- | --- | --- | --- |
| Mm1 | 64 | 16 | 8 | 8 | 4 |
| Mm2 | 64 | 32 | 8 | 8 | 8 |
| Mm3 | >512 | >1024 | <0.5 | 64 | 8 |
| X | 32 | 8 | <0.5 | 8 | 4 |

**REFERENCES**

1. Elbehery, A. H. A., Aziz, R. K. & Siam, R. Antibiotic Resistome: Improving Detection and Quantification Accuracy for Comparative Metagenomics. *Omi. A J. Integr. Biol.* **20,** 229–238 (2016).

2. Han, J., Sahin, O., Barton, Y.-W. & Zhang, Q. Key role of Mfd in the development of fluoroquinolone resistance in Campylobacter jejuni. *PLoS Pathog.* **4,** e1000083 (2008).

3. Savery, N. J. The molecular mechanism of transcription-coupled DNA repair. *Trends Microbiol.* **15,** 326–33 (2007).

4. Naas, T. & Nordmann, P. Analysis of a carbapenem-hydrolyzing class A beta-lactamase from Enterobacter cloacae and of its LysR-type regulatory protein. *Proc. Natl. Acad. Sci. U. S. A.* **91,** 7693 (1994).
